# Speech timing cues reveal deceptive speech in social deduction board games

**DOI:** 10.1371/journal.pone.0263852

**Published:** 2022-02-11

**Authors:** Ziyun Zhang, Carolyn McGettigan, Michel Belyk

**Affiliations:** 1 Department of Speech, Hearing and Phonetic Sciences, University College London, London, United Kingdom; 2 Department of Psychology, Edge Hill University, Ormskirk, United Kingdom; University of Padova, ITALY

## Abstract

The faculty of language allows humans to state falsehoods in their choice of words. However, while what is said might easily uphold a lie, *how* it is said may reveal deception. Hence, some features of the voice that are difficult for liars to control may keep speech mostly, if not always, honest. Previous research has identified that speech timing and voice pitch cues can predict the truthfulness of speech, but this evidence has come primarily from laboratory experiments, which sacrifice ecological validity for experimental control. We obtained ecologically valid recordings of deceptive speech while observing natural utterances from players of a popular social deduction board game, in which players are assigned roles that either induce honest or dishonest interactions. When speakers chose to lie, they were prone to longer and more frequent pauses in their speech. This finding is in line with theoretical predictions that lying is more cognitively demanding. However, lying was not reliably associated with vocal pitch. This contradicts predictions that increased physiological arousal from lying might increase muscular tension in the larynx, but is consistent with human specialisations that grant *Homo sapiens sapiens* an unusual degree of control over the voice relative to other primates. The present study demonstrates the utility of social deduction board games as a means of making naturalistic observations of human behaviour from semi-structured social interactions.

## Introduction

Deception is a widespread and fundamental aspect of communication, that has been observed across a wide range of species. While the flexibility of human communication through the faculty of language provides particular opportunities for deception, an anthropocentric perspective on lying may be overly limiting. It is a general principle that systems of communication are only adaptive if they are on average honest. However, the interests of the individual often incentivise deception [1,2]. Usually the honesty-on-average of communication comes from some constraint on the effectiveness of deception, such as the physical, physiological, or cognitive limitations of the deceiver [3,4]. In the context of human speech, what is said might easily uphold a lie, but *how* it is said may reveal deception.

Several classes of theories have attempted to describe how listeners are able to detect deceptive speech in spite of the intentions of the speaker [5,6]. Of these, two make concrete and falsifiable predictions about limitations on the speaker’s ability to control the acoustical signal of speech that may render attempted deceptions detectable. Cognitive load accounts of deception posit that deception requires more cognitive processing power than truth-telling, leading to more and longer silent pauses and filled pauses (e.g., “uh”, “um”) in speech [6,7]. This is consistent with the standard doctrine in cognitive psychology that increased cognitive processing causes delays in response times [8,9] including in speech [10,11]. Even deception without speech, such as in responding dishonestly on personality questionnaires, yields longer response times than responding honestly [12–14]. Arousal accounts of deception highlight that lying may cause greater anxiety [15], which increases muscle tension throughout the body including the muscles of the larynx that control the voice [16,17], resulting in higher vocal pitch [18,19].

While there is evidence that increased pausing or increased vocal pitch may mark deceptive speech, these findings are inconsistent [20–23], and effect sizes are correspondingly modest [5,24]. However, in many of these studies participants had limited motivation to lie successfully, or the context in which they lied had little relation to real-life communication. For example, in many cases participants were told what to lie about and when, which may undermine the cognitive load and/or arousal mechanisms that are putatively being tested. Indeed, liars who are more strongly motivated to deceive successfully are more likely to exhibit behaviours that reveal their lie [25].

Motivated and ecologically valid deception is difficult to record in a laboratory setting. Some experiments have used monetary incentives to motivate successful lying [26,27]. Others have used the natural motivation of students whose professional interests may require deception, such as the development of bedside manner in nursing students [28]. Some exceptional experiments have analysed recordings of police interviews based on statements which were eventually verified to be dishonest [29–31]. Such studies provide maximal ecological validity from genuine and high-stakes instances of lying, but are necessarily lacking in experimental control and are limited to small samples of individuals who may not be representative of the broader population. Hence, there is a need for scalable approaches to developing ecologically valid deception corpora in which speakers with sufficiently strong motivation will lie of their own volition.

## Methods

We observed the natural speech of players of a popular social deduction board game, in which players are assigned roles that either promote honest or dishonest interactions but otherwise engage in free conversation. In these sessions, players made claims, asked each other questions, told lies, or spoke truths on their own initiative. Audio recordings from game sessions were acoustically analyzed to test for markers of deception as predicted by the cognitive load and arousal theories of deception.

### Participants

Fourteen adults (9 males and 5 females) were recruited from a London-based board game group holding meetings online. Thirteen participants were native speakers of English. All players indicated that they had moderate to extensive prior experience with the game. The research was approved by the Department of Speech, Hearing and Phonetic Science, University College London [SHaPS-2019-CM-030]. Participants provided written informed consent for their recordings to be analysed for research purposes. As a token of our appreciation, all participants were entered into a draw for one of two £50 vouchers.

### The game

We recorded natural utterances from speakers of a board gaming community as they played the popular social deduction game, *Secret Hitler* (https://www.secrethitler.com/, licensed BY-NC-SA 4.0). It should be noted that *Secret Hitler* does not advocate or endorse authoritarian politics, but rather it is a caution against the insidious creep of fascism. In the game, players are assigned roles that either induce honest or dishonest interactions as they talk to each other and make decisions about whom to trust.

Secret Hitler is played with 5–10 players who are divided into two teams: Liberals and Fascists. One member of the Fascist team also has the secret role of “Hitler”. The Liberals are in the majority, but they do not know the role of any other player. The Fascists are in the minority but know the identities of all other players, with the exception of the player with the role of “Hitler”, who is also uninformed. In each round of play, the players elect a President and a Chancellor. The President draws three policy tiles and secretly passes two of these to the Chancellor, who then chooses one of these policies to enact. These policies are either Liberal or Fascist and the first team to pass a predetermined number of their own policies wins. Occasionally there are opportunities to eliminate a player, and the Liberals win if they eliminate “Hitler”. Conversely, the Fascists win if “Hitler” is elected Chancellor after they have passed at least three fascist policies. In games with 5–6 players, “Hitler” also has complete knowledge along with The Fascists.

The asymmetrical design of this game motivates Liberals to be truthful because their majority could force favorable outcomes if they gather sufficient information. Conversely, Fascists are motivated to be deceptive, to sow distrust among the Liberals, and to keep the role of “Hitler” from being discovered. Therefore, to win the game, the Fascists usually lie to hide their identities and gain the trust of the Liberals, while the Liberals do not have reason to lie in most cases.

### Procedure

We made audio recordings of the speakers across multiple gameplay sessions held online. Each game session consisted of approximately 25–60 minutes of continuous and free conversation in which players made claims, asked each other questions, lied, or told the truth on their own initiative. Players contributed to multiple iterations of the game, with roles randomly assigned across sessions. We identified phrases that were likely to be said often as both truths and lies (e.g. “I am a Liberal” or “I know they are a Fascist”) for acoustical analysis. A total of 116 lies and 350 truthful utterances were recorded across 13 iterations of play (See S1 File for further details). Acoustical measurements were taken from each utterance to assess the presence and duration of silent and filled pauses, as well as voice cues including the mean and standard deviation of fundamental frequency (the acoustic correlate of voice pitch).

### Ground truth identification of lies

The structure of this game leads players to frequently produce certain classes of statements. Four categories of sentences were identified as being likely to occur often as either a truth or a lie: A) utterances about the type of tiles a player drew (e.g., “I picked up two Liberals and one Fascist, and I discarded a Liberal”), B) utterances about one’s own identity (e.g., “I’m a Liberal”), C) utterances about others’ identities (e.g., “I know she’s Fascist”), and D) utterances about the current situation (e.g., “we’re in a good condition now”).

After the outcome of each game was decided and all secret roles were revealed, it was possible to know the truth value of statements of these categories. Three participants produced no confirmed lies but 4–25 confirmed truths. The remaining participants produced 1–32 confirmed lies and 7–91 confirmed truths. Only one participant produced more confirmed lies than confirmed truths (32 and 18, respectively). See S1 Table in S1 File for the distribution of lie categories.

### Interface and communications

**Fig 1:**
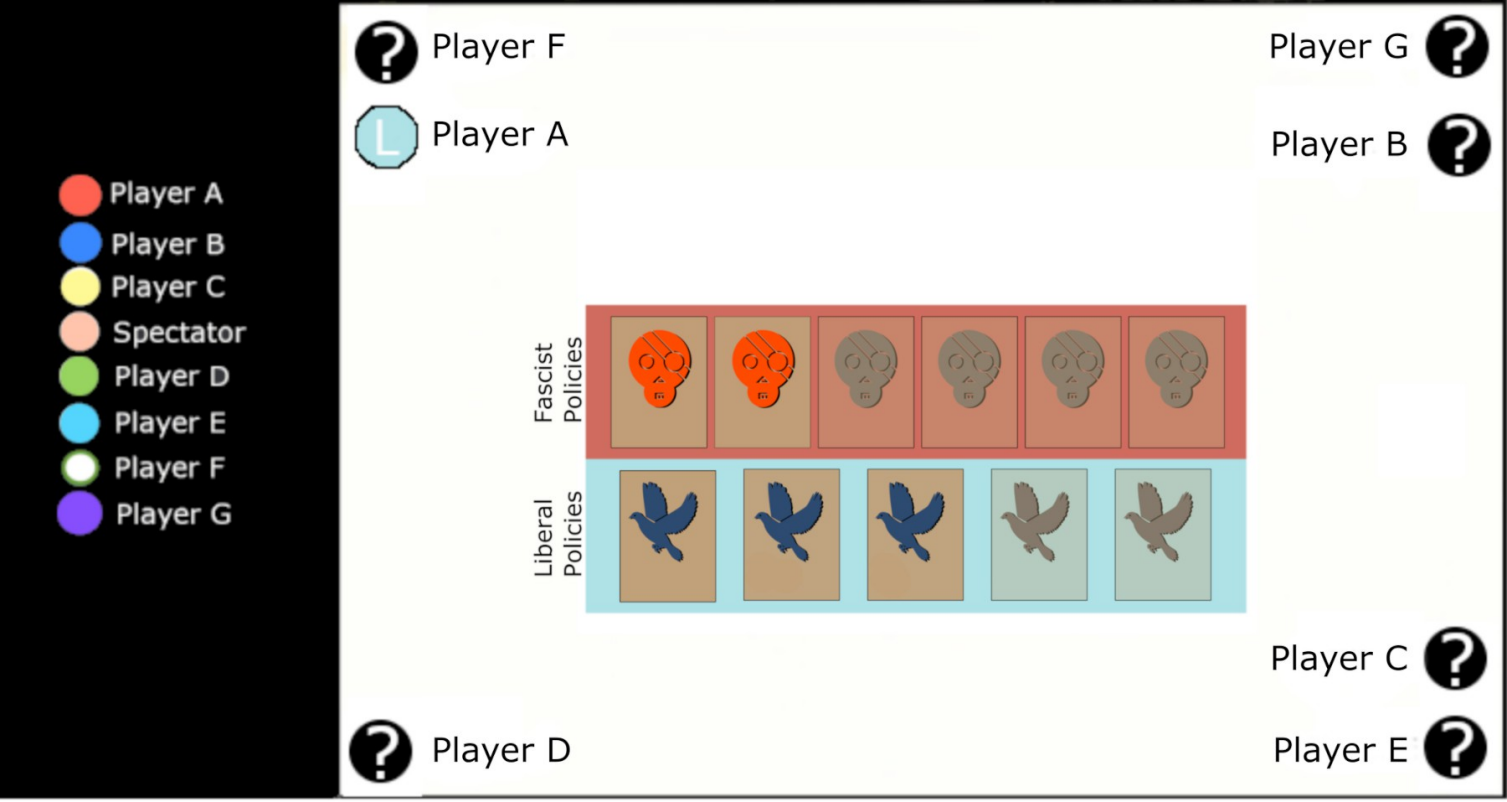
Schematic of Communications (left) and game (right) interfaces as viewed from the perspective of Player A. This participant was playing as a Liberal (blue team) who does not know the roles of any other player. A visual cue (green circle) indicates that Player F is presently speaking. In the scenario depicted, the Fascist team has passed 2 of the 6 policies they need to win, while the liberals have passed 3 of the 5 policies that they need to win.

Secret Hitler was played online with a virtual table (https://secret.ethanl.ee/). Before each Secret Hitler game, players who intended to join the game formed a temporary audio channel on Discord (https://discord.com/) which formed an open channel of communication between all players and was recorded by the experimenter with the consent of the players. In this system, the players’ avatars included a visual cue that provides an objective means of determining which player was speaking in order to ensure that utterances were attributed to the correct speaker (see Fig 1).

### Measurement of speech acoustics

Each audio recording was viewed and edited with an annotated TextGrid in Praat [32] (Version 6.1.16, in MacOS Catalina 10.15). Clips corresponding to the four pre-defined classes of phrases were excised from the full audio recordings, and labelled with the participant ID and the truthfulness of the statement. A semi-automated Praat script was used to extract acoustic measurements from each clip, including mean vocal pitch (f_0_-mean), standard deviation of vocal pitch (f_0_-SD)_,_ total sounding duration, minimum sounding duration, maximum sounding duration, total silent duration and the number of silent pauses of each sound clip. Silent and sounding periods were automatically detected with a silence threshold -25.0 dB, minimum silent period 0.1 seconds and minimum sounding period 0.05 seconds. All sounding and silent detections were manually corrected by visual inspection. Filled pauses were also detected manually as syllables that were devoid of semantic or syntactic content.

### Statistical analysis

Acoustic measures of f_0_-mean and f_0_-SD were analysed using linear mixed models (LMMs) [33] in R [34]. Statistical significance was assessed by comparing full models to null models that lacked the focal predictor, following type III sums of squares. Models that included random slopes of Truthfulness across participants frequently led to singular fits and therefore only random intercepts were retained. The standard assumptions of multilevel modelling were tested: Initial model fits for f_0_-mean and f_0_-SD measures suggested severe violations of normality of residuals and of homoscedasticity. Hypotheses for f_0_ measurements were therefore tested by parametric bootstrapping with 1000 simulations, in order to assess the null hypothesis against an empirical null distribution rather than poorly matched theoretical distributions [35]. Fixed-effect coefficients for these models show the estimated change in f_0_-mean or f_0_-SD from truths to lies.

An examination of the distribution of responses for the silent and filled pause duration measurements revealed poor correspondence to any standard distribution. However, these responses strongly resembled a gamma distribution with the addition of a large number of zeros introduced by instances in which no pauses were observed in the data (i.e., pause durations of 0 ms in perfectly fluent speech). These data were therefore analysed following the template model builder approach in which complex distributions are accommodated by parametrically combining simpler distributions [36]. Hence, we constructed generalized linear mixed models which combined one parameter following a gamma distribution to capture pause durations spanning the theoretical range of all positive values, and a separate zero inflation term to model the presence of zeros, which could otherwise not be modelled by a gamma distribution. These models satisfied assumptions for both the distribution of residuals and homoscedasticity. Leverage analyses were conducted to assess models for outliers (see S1.1-S1.4 Figs in S1 File). The non-native speaker of English was not among the outlier candidates.

In addition to improving the statistical validity of the models, this approach provided a means for conducting separate statistical tests on the presence of pauses using the zero-inflation term (by comparing the performance of models which were free to allow a different number of zero responses for lies versus truths against models which were not) and the duration of pauses using the gamma term (by comparing the performance of models which were free to allow different durations of pauses for lies versus truths against models which were not). See model specification in Eq 1.


$$\begin{array}{l} \mathrm{Formula}:\;\mathrm{Acoustic}\_\mathrm{measurement}∼1+\mathrm{Truthfulness}+(1|\mathrm{Participant}\_\mathrm{ID})\\ \mathrm{Zero}\;\mathrm{inflation}:\;∼1+\mathrm{Truthfulness}\\ \mathrm{Family}:\\ \mathrm{ziGamma}(\mathrm{link}=\log) \end{array}$$ Eq (1)

Fixed effect coefficients for these models show the expected change in log-pause duration and the log-ratio of pause absences from truths to lies. The results are reported with exponentiated coefficients so that they may be interpreted on a linear scale, rather than a logarithmic scale. To further facilitate interpretation, we have inverted the sign of the zero-inflation coefficients so that they show the increase in the presence of pauses for lying rather than a decrease in the absence of pauses for truths. These statements are equivalent, but the former is more easily interpreted.

## Results

**Fig 2:**
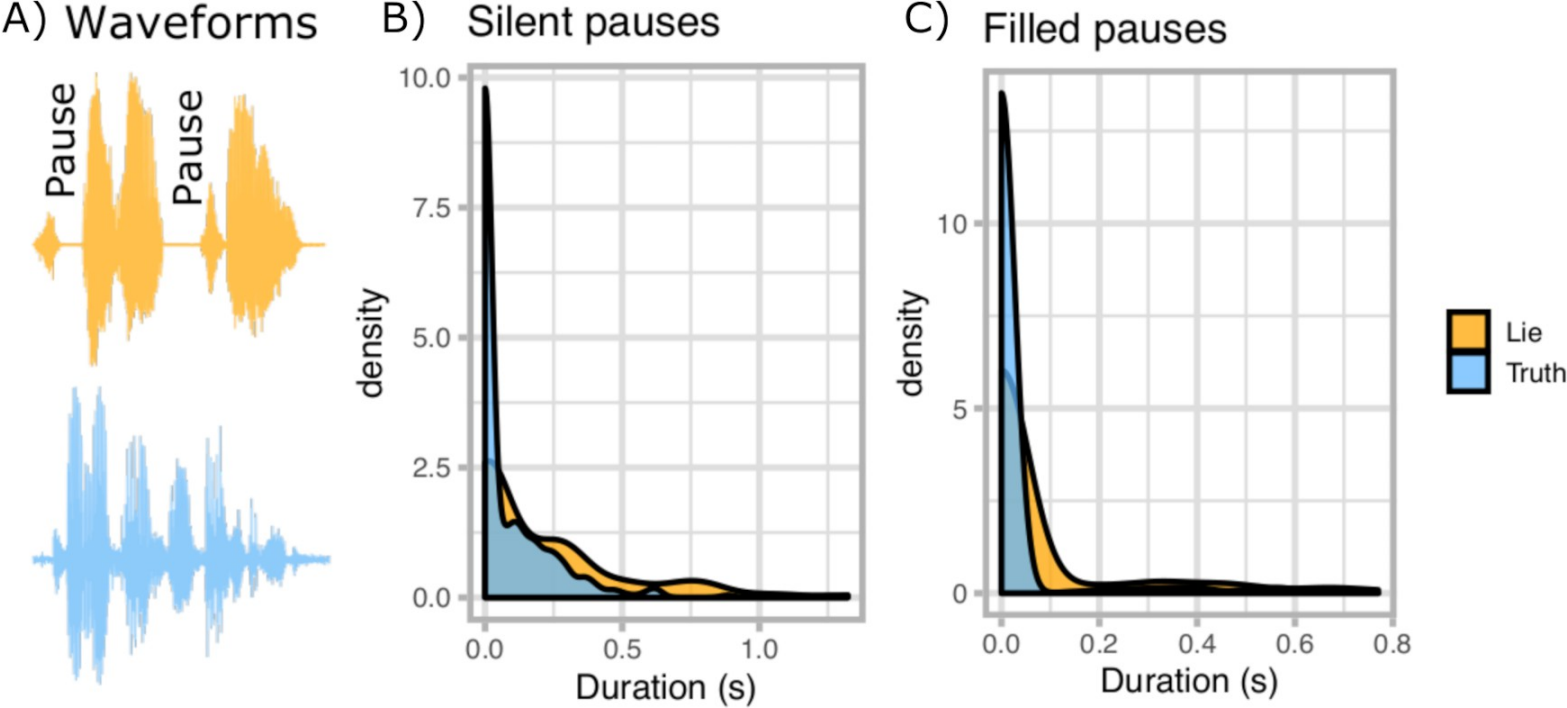
A) Example waveforms for one deceptive (top) and one honest (bottom) speech utterance. B) Density plot showing the distribution of the durations of silent pauses and C) filled pauses for lies (orange) and truths (blue). Note the abundance of truthful utterance without pauses (i.e. with pause durations of 0 ms).

Changes in vocal acoustics when players lied were analysed using generalised linear mixed effects models [36]. Deceptive speech was more likely to contain silent pauses (*χ*^2^(1) = 11.53, p < 0.001, estimate = 0.73, CI = [0.31–1.16]), where the pauses had a longer mean duration (*χ*^2^ (1) = 25.47, p < 0.001, estimate = 0.56, CI = [0.34–0.78]) than those in truthful speech (see Fig 2). This corresponds to lies being twice as likely to contain silent pauses (exp(0.73) = 2.08) which are 76% longer (exp(0.56) = 1.76 times longer pauses).

Deceptive speech was also more likely to contain filled pauses (e.g. “umm” or “uhhh”; *χ*^2^ (1) = 9.13, p = 0.003, estimate = 1.06, CI = [0.39–1.73]) to such an extent that too few filled pauses were observed in truthful statements (5.9%) to support a meaningful test of filled pause durations. This corresponds to lies being nearly three times as likely to contain filled pauses (exp(1.06) = 2.88).

**Fig 3:**
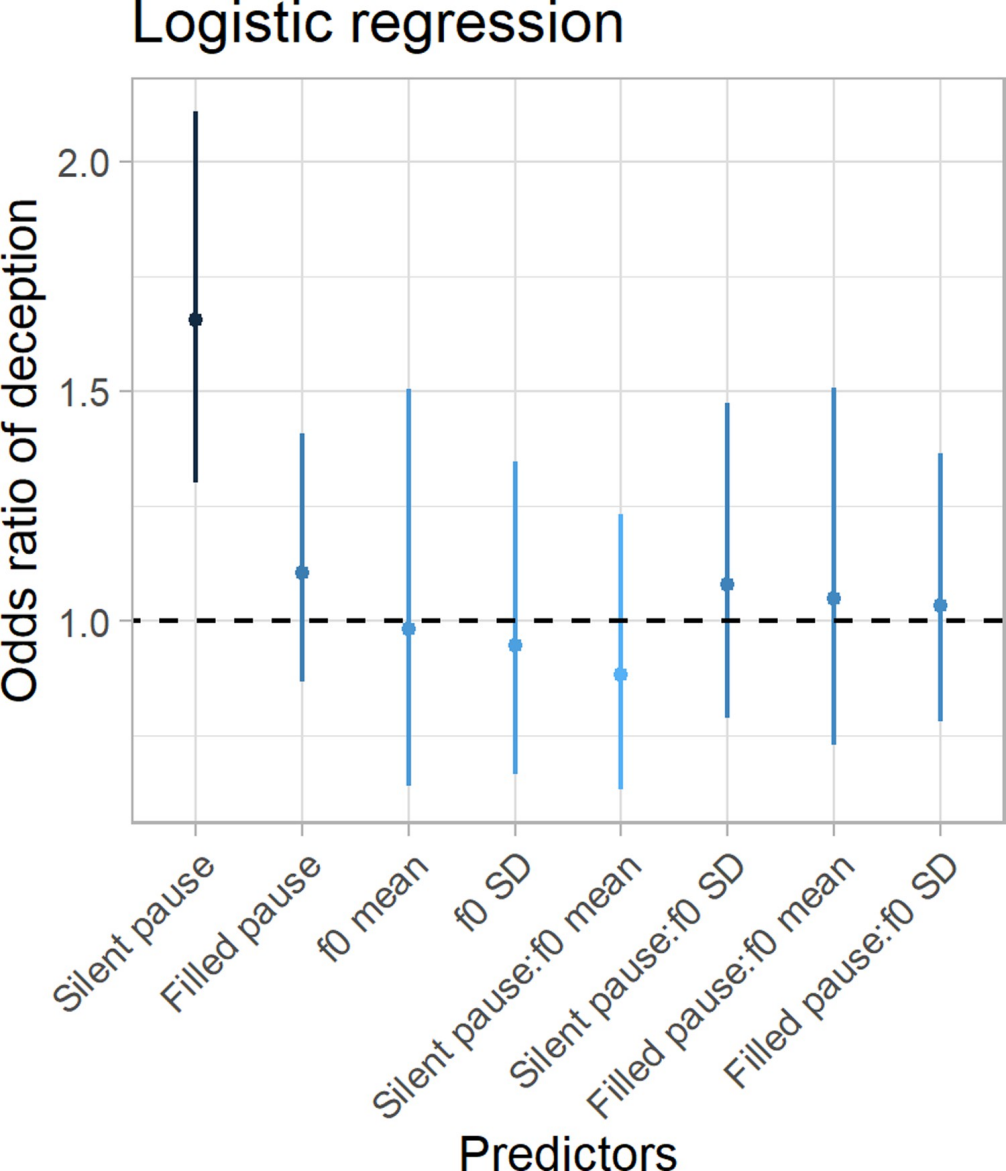
Estimates from logistical regression. The y-axis plots the odds-ratio: The increase in the odds that a statement is deceptive for every increase of 1 SD in each predictor variable. A 1 SD increase in the mean duration of silent pauses indicates that a statement has 1.66 times the odds of being deceptive. The x-axis reports all predictors that were included in the logistical regression model with interactions indicated by full colons (:). Line ranges indicate 95% confidence intervals.

In contrast, lying had no measurable influence on mean vocal pitch (*χ*^2^ = 0.38, p = 0.53, estimate = - 2.43, CI = [-10.15–5.79]) or its standard deviation (*χ*^2^ = 0.12, p = 0.74, estimate = -1.73, CI = [-11.46–7.83]; see S1.5 Fig in S1 File). Supplementary analyses using logistic regression to predict deception from acoustical parameters (see S1 File for further details) yielded similar results with no interactions between pause and pitch measures (see Fig 3).

## Discussion

We used a novel social deduction board game paradigm to search for acoustic markers of dishonest speech. By leveraging the game’s tendency to generate freeform conversations about structured events, this paradigm allowed us to produce ecologically valid recordings of deceptive speech from a larger cohort than would typically be feasible. Furthermore, this approach naturally constrained the scope of topics about which speakers were dishonest, preserving a degree of experimental control that would otherwise have had to be sacrificed.

We observed strong evidence that dishonest speech contains more frequent and longer pauses. Unlike truths, the contents of a lie are not necessarily plausible and liars are faced with the additional cognitive burden of checking that their statements do not contradict information that is already known to the listener. Pauses may be signs of increased cognitive demand as they give the speaker additional time for problem-solving using fixed cognitive capacity [37]. Importantly, these cues are likely inserted into deceptive speech due to mechanisms beyond the speaker’s control. Hence, they may mark deceptive statements despite the speaker’s dishonest intentions.

We observed no evidence that dishonest speech contains higher or more variable vocal pitch. Indeed, effect estimates were sufficiently small that if they proved reliable with a larger sample they would remain below the threshold of human auditory discrimination [38]. This is inconsistent with a view that lying may be marked by cues associated with greater arousal, anxiety, or excitement [39]. It is possible that the low-stakes context of the board game produced little anxiety, although the fact that players elected to participate in these games as a leisure activity supports a reasonable assumption that they found the games exciting and were motivated to win. Alternatively, the exceptional control that humans have over the pitch of their voice may provide the means for liars to mask voice cues that might otherwise reveal a lie. Humans have well-documented neural specialisations for the control of vocal pitch which are either absent or incipient in other primates [40]. Liars may therefore be able to inhibit vocal pitch cues that would undermine their intention to deceive.

These findings support the view that factors outside of the liar’s control—such as strains on limited cognitive processing power [27]—can belie dishonest speech. The existence of such constraints on deception is consistent with observations from communicative systems across a broad range of species and taxa. Communicative systems that have evolved in non-human animals are primarily maintained by being cues of quality over which the animal has no control, by being too costly or too strongly constrained by the animal’s anatomy to fake, or by risking reputation, reprisal, and the ability to influence the behaviour of others if the deception is discovered [2–4]. For example, spiders fighting over territory have their body weights, a primary predictor of conflict outcome, faithfully communicated through the vibrations carried by the web that is being fought over [41]. In mammals, acoustic cues in the voice can also indicate body size [42,43]. While some species are able to exaggerate their vocal size, anatomical constraints keep these signals relatively honest [44]. Some primates have been observed to issue anti-predator alarm calls deceptively during food contests, but at the expense of conspecifics becoming less responsive to their alarm calls in future [45].

For behavioural ecology accounts of communication in non-human animals, ecological validity is naturally an item of consideration. For psychological and linguistic experiments in humans, it is often expedient for researchers to rely on the cooperation of the participants to exhibit the behaviour which is under study. However, this expedience is often won at the expense of ecological validity and not necessarily at a reasonable rate of exchange. The difficulty of studying deceptive communication in an ecologically valid context is further compounded by the probable uncooperativeness of speakers who are choosing to deceive. The present study demonstrates the utility of social deduction board games as a means of making naturalistic observations of human behaviour from semi-structured social interactions. Future research can apply this methodology at a larger scale to further understand the mechanisms that lead lies to be detectable, or not, in naturalistic speech.

### Limitations

The present study was based on a relatively small sample of speakers engaged in spontaneous behaviour under relatively uncontrolled conditions. While these limitations are an expected cost of observing behaviour beyond the laboratory, they may impact the interpretation of the results. Participants communicated virtually through an audio call from their homes. Consequently, the researchers were unable to control the participants’ auditory environment or their audio recording and playback equipment. Likewise, as the game incentivises deception but never mandates whether, how often, or in what way players deceive, we observed considerable variation in the number of deceptive statements recorded per participant. Some control is retained by the fact that participants are assigned roles that incentivise either honesty or dishonesty, potentially limiting the confounding effects of the personal characteristics of the speaker. Furthermore, as participants communicated through audio only, deception may have been rendered easier by obscuring other channels of communication, such as hand gestures, that may provide further cues to deception either on their own or in combination with speech [46]. Overall, while our approach does not have the ecological validity of examining high-risk deception *in situ*, as in police interviews, it does strike a desirable balance with experimental control providing benefits such as objective identification of deceptions, random assignment, and scalability.

## Conclusion

The number and duration of silent and filled pauses were associated with deceptive speech consistent with the cognitive load account of deception. Notably, this account is consistent with established frameworks for understanding deceptive signalling in all other species–namely that stable systems of communications are ones in which the limitations of the signaller constrain their ability to deceive. Social deduction board games provide a plausible means of producing well-motivated, ecologically valid, semi-structured conversations.

## Supporting information

S1 File Supplementary methods, analyses, tables, and figures. (DOCX)

S2 File Open data. (CSV)

S3 File Open code. (R)

S4 File Game rules. (PDF)
