# Supplementary material for: Speech timing cues reveal deceptive speech in social deduction board games
Source: PLoS One. 2022 Feb 11;17(2):e0263852. doi: 10.1371/journal.pone.0263852 (PMC8836341; doi:10.1371/journal.pone.0263852)
Supplement: S1 File — (DOCX) [file pone.0263852.s001.docx]

**ETHNICS AND SUBJECT DETAILS**

***Ethics statement***

The research was approved by the Department of Speech, Hearing and Phonetic Science, University College London [SHaPS-2019-CM-030]. Participants provided informed consent for their recordings to be analyzed, via the online platform Gorilla (https://gorilla.sc/). As a token of our appreciation, all participants were entered into a draw for one of two £50 vouchers. Secret Hitler is licensed under Creative Commons BY-NC-SA 4.0.

***Participants***

Fourteen adults (9 males and 5 females) were recruited from a London-based boardgame group holding meetings online. Thirteen participants were native speakers of English. All players indicated that they had moderate to extensive prior experience with the game.

**METHODS DETAILS**

***Secret Hitler***

Secret Hitler (<https://www.secrethitler.com/>) is a social deduction game for 5-10 people. Players are divided into two teams: The Liberals and the Fascists. One member of the Fascist team also has the secret role of “Hitler”. The Liberals are in the majority, but they do not know the role of any other player. The Fascists are in the minority but know the identities of all other players, with the exception of the player with the role of “Hitler”, who is also uninformed. In each round of play, the players elect a President and a Chancellor. The President draws three policy tiles and secretly passes two of these to the Chancellor, who then chooses one of these policies to enact. These policies are either Liberal or Fascist and the first team to pass a predetermined number of their policies wins. Occasionally there are opportunities to eliminate a player, and the Liberals win if they eliminate “Hitler”. Conversely, the Fascists win if “Hitler” is elected Chancellor after they have passed at least three fascist policies. In games with 5-6 players, “Hitler” also has complete knowledge along with The Fascists. See S4 for a full copy of the rules.

The asymmetrical design of this game motivates Liberals to be truthful since their majority could force favorable outcomes if they gather sufficient information. Conversely, Fascists are motivated to be untruthful, to sow distrust among the Liberals, and to keep the role of “Hitler” from being discovered. Therefore, to win the game, the Fascists usually lie to hide their identities and gain the trust of the Liberals, while the Liberals do not have reason to lie in most cases. The structure of this game leads players to frequently produce certain classes of statements. Four categories of sentences were identified as being likely to occur often as either a truth or a lie: A) utterances about the type of tiles a player drew (e.g., “I picked up two Liberals and one Fascist, and I discarded a Liberal”), B) utterances about one’s own identity (e.g., “I’m a Liberal”), C) utterances about others’ identities (e.g., “I know she’s Fascist”), and D) utterances about the current situation (e.g., “we’re in a good condition now”). After the outcome of each game was decided and all secret roles were revealed, it was possible to know the truth value of statements of these categories. Three participants produced no confirmed lies but 4-25 confirmed truths. The remaining participants produced 1-32 confirmed lies and 7-91 confirmed truths. Only one participant produced more confirmed lies than confirmed truths (32 and 18, respectively). See table S1 for the distribution of lie categories.

Secret Hitler was played online with a virtual table. Before each Secret Hitler game, players who intended to join the game formed a temporary audio channel on Discord (<https://discord.com/>) for communication. In this system, the players’ avatars provide a visual cue as an objective means of determining which player was speaking, which ensured that utterances where correctly attributed to speakers.

***Measurement of speech acoustics***

Each audio recording was viewed and edited with an annotated TextGrid in Praat^1^ (Version 6.1.16, in MacOS Catalina 10.15). Clips corresponding to the four pre-defined classes of phrases were excised from the full audio recordings, and labelled with the participant ID and the truthfulness of the statement. A semi-automated Praat script was used to extract acoustic measurements from each clip, including mean vocal pitch (f_0_-mean), standard deviation of vocal pitch (f_0_-SD)_,_ total sounding duration, minimum sounding duration, maximum sounding duration, total silent duration and the number of silent pauses of each sound clip. Silent and sounding periods were automatically detected with a silence threshold -25.0 dB, minimum silent period 0.1 seconds and minimum sounding period 0.05 seconds. All sounding and silent detections were manually corrected by visual inspection. Filled pauses were also detected manually as syllables that were devoid of semantic or syntactic content.

**STATISTICAL DATA ANALYSIS**

Acoustic measures of f_0_-mean and f_0_-SD were analyzed using linear mixed models (LMMs)^2^ in R^3^. LMM took the form:

Formula: *Acoustic measurement ~ 1 + Truthfulness + (1 | Participant.ID)*

Statistical significance was assessed by comparing full models to null models that lacked the focal predictor, following type III sums of squares. Models that included random slopes of Truthfulness across participants frequently led to singular fits and therefore only random intercepts were retained. The standard assumptions of multilevel modelling were tested: Initial model fits for f_0_-mean and f_0_-SD measures suggested severe violations of normality of residuals and of homoscedasticity. Hypotheses for f_0_ measurements were therefore tested by parametric bootstrapping with 1000 simulations, in order to assess the null hypothesis against an empirical null distribution rather than poorly matched theoretical distributions^4^. Fixed-effect coefficients for these models show the estimated change in f_0_-mean or f_0_-SD from truths to lies.

An examination of the distribution of responses for the silent and filled pause duration measurements revealed poor correspondence to any standard distribution. However, these responses strongly resembled a gamma distribution with the addition of a large number of zeros introduced by instances in which no pauses were observed in the data (i.e., pause durations of 0 ms in perfectly fluent speech). These data were therefore analyzed following the template model builder approach in which complex distributions are accommodated by parametrically combining simpler distributions^5^. Hence, we constructed generalized linear mixed models which combined one parameter following a gamma distribution to capture pause durations spanning the theoretical range of all positive values, and a separate zero inflation term to model the presence of zeros, which could otherwise not be modelled by a gamma distribution. These models satisfied assumptions for both the distribution of residuals and homoscedasticity.

In addition to improving the statistical validity of the models, this approach provided a means for conducting separate statistical tests on the presence of pauses using the zero-inflation term (by comparing the performance of models which were free to allow a different number of zero responses for lies versus truths against models which were not) and the duration of pauses using the gamma term (by comparing the performance of models which were free to allow different durations of pauses for lies versus truths against models which were not). These models too the following form:

Formula: *Acoustic measurement ~ 1 + Truthfulness + (1 | Participant.ID)*

Zero inflation: *~ 1 + Truthfulness*

Family: ziGamma(link=log)

Fixed effect coefficients for these models show the expected change in log-pause duration and the log-ratio of pause absences from truths to lies. In the main text of the manuscript we exponentiate these coefficients to facilitate interpretation on a linear scale, rather than a logarithmic scale. To further facilitate interpretation, we have inverted the sign of the zero-inflation coefficients so that they show the increase in the presence of pauses for lying rather than the decrease in the absence of pauses for truths. These statements are equivalent, but the former is more easily interpreted.

***Assessment of potential outliers***

Cooks distance is am easure derived from iteratively refitting models with individual participants omitted in order to assess their influence on model fits. Figures S1.1-S1.4 plot parameter estimates and cooks distnaces per participant. While this measure is not intended as a replacement for the analysts judgement it provides a means of identifying data points that merit further consideration. The participants with the largest cooks distances are labelled in each plot. Notably the sole non-native speaker of English was not flagged for further inspection in any model.

***Logistic regression***

In response to a reviewer’s inquiry as to whether the combination of pause and pitch measurements may be informative, even if pitch alone is not, we conducted a post-hoc logistic regression analysis.

We can constructed a logistic regression model to predict binary truth/lie status of statements from fixed effects of silent duration, filled pause duration fo_mean, and f0 SD with random intercepts of participant. A fully crossed model that included all possible interaction terms was too complex to fit from these data. We therefore selected four interaction terms that tests the reviewers hypothesis (i.e., silent pauses with mean f0, silent pauses with f0SD, filled pauses with mean f0, filled pauses with f0SD).

The model took the form:

Truth.Lie ~ 1 + silent_duration_ave + filled_duration_ave

+ f0_mean + f0_SD

+ silent_duration_ave:f0_mean + silent_duration_ave:f0_SD

+ filled_duration_ave:f0_mean + filled_duration_ave:f0_SD

+ (1|Participant.ID)

No interaction approached significance with test statistics (Z) ranging between -0.7 and 0.5 and p-values ranging from 0.46 to 0.82. Only the main effect of silent pause duration was significant (Z=4.1, p<0.001) which is consistent with our primary analysis in which we observed that filled pauses occurred too seldomly during truthful statements to permit a meaningful test.

Fixed effects:

Estimate Std. Error z value Pr(>|z|)

(Intercept) -1.57882 0.32205 -4.902 9.46e-07 ***

silent_duration_ave 0.50448 0.12329 4.092 4.28e-05 ***

filled_duration_ave 0.10012 0.12313 0.813 0.416

f0_mean -0.01746 0.21735 -0.080 0.936

f0_SD -0.05381 0.17929 -0.300 0.764

silent_duration_ave:f0_mean -0.12428 0.16994 -0.731 0.465

silent_duration_ave:f0_SD 0.07574 0.15974 0.474 0.635

filled_duration_ave:f0_mean 0.04879 0.18438 0.265 0.791

filled_duration_ave:f0_SD 0.03197 0.14192 0.225 0.822

---

Signif. codes: 0 ‘***’ 0.001 ‘**’ 0.01 ‘*’ 0.05 ‘.’ 0.1 ‘ ’ 1

**References**

1. Boersma, P., and Weenink, D. (2016). Praat: Doing phonetics by computer.

2. Bates, D., Maechler, M., Bolker, B., and Walker, S. (2015). Fitting linear mixed-effects models using lme4. J. Stat. Softw. *67*, 1–48.

3. R Core Team (2019). R: A language and environment for statistical computing.

4. Halekoh, U., and Højsgaard, S. (2014). A Kenward-Rogers Approximation and Parametric Bootstrap Methods for Tests in Linear Mixed Models - The R Package pbkrtest. J. Stat. Softw. *59*, 1–30.

5. Brooks, M.E., Kristensen, K., van Benthem, K.J., Magnusson, A., Berg, C.W., Nielsen, A., Skaug, H.J., Maechler, M., and Bolker, B.M. (2017). glmmTMB: Balances speed and flexibility among packages for zero-inflated generalized linear mixed modeling. R J. *9*, 278–400.


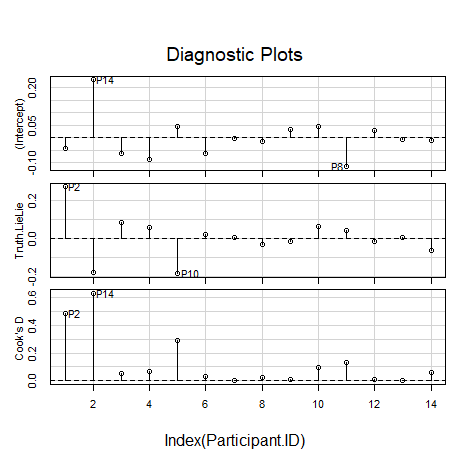


FigureS1.1: Cooks distances for model of silent pauses.


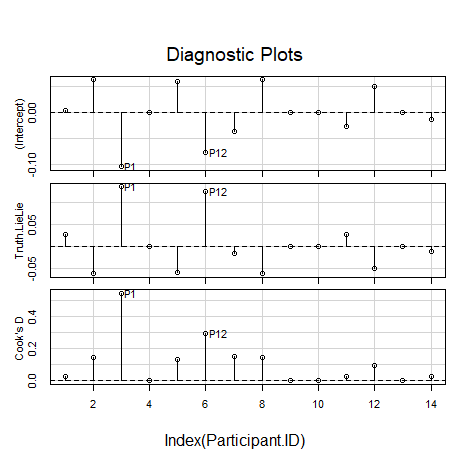


FigureS1.2: Cooks distances for model of filled pauses.


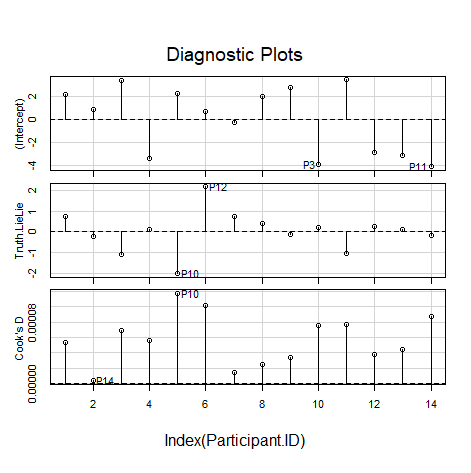


FigureS1.3: Cooks distances for model of mean f0.


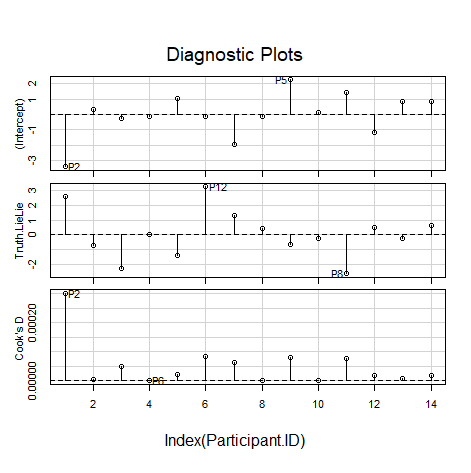


FigureS1.4 Cooks distances for model of f0 SD.


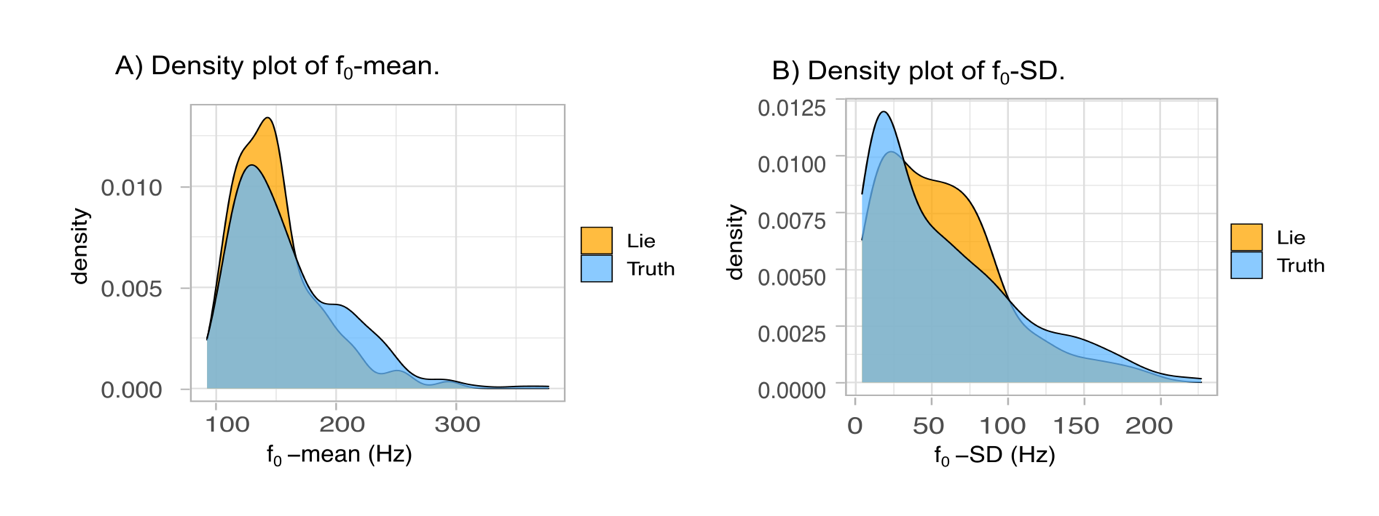


Figure S1.1:  A) Density plot of f_0_-mean and B) f_0_-SD for lies and truths. There was no detectable difference in either voice pitch measurement between truths and lies.

| \| Utterance types \| \| --- \|   Participant | Lies | | | | Truths | | | |
| --- | --- | --- | --- | --- | --- | --- | --- | --- | --- |
|  | A | B | C | D | A | B | C | D |
| P1 | 2 | 0 | 28 | 2 | 14 | 0 | 5 | 0 |
| P2 | 2 | 3 | 15 | 4 | 32 | 2 | 43 | 13 |
| P3 | 2 | 1 | 3 | 1 | 12 | 0 | 8 | 6 |
| P4 | 1 | 0 | 0 | 0 | 9 | 1 | 2 | 0 |
| P5 | 0 | | | | 14 | 7 | 4 | 0 |
| P6 | 0 | | | | 4 | 0 | 0 | 0 |
| P7 | 3 | 0 | 0 | 0 | 6 | 0 | 9 | 1 |
| P8 | 2 | 0 | 1 | 0 | 10 | 0 | 6 | 2 |
| P9 | 0 | | | | 6 | 3 | 8 | 1 |
| P10 | 5 | 1 | 12 | 0 | 13 | 5 | 12 | 2 |
| P11 | 0 | 0 | 4 | 0 | 5 | 0 | 4 | 0 |
| P12 | 1 | 0 | 12 | 3 | 26 | 0 | 11 | 4 |
| P13 | 0 | 0 | 1 | 0 | 5 | 1 | 1 | 0 |
| P14 | 2 | 1 | 4 | 0 | 15 | 2 | 13 | 3 |

Table S1: The number of confirmed lies and confirmed truths by participant. A) utterances about the type of tiles a player drew (e.g., “I picked up two Liberals and one Fascist, and I discarded a Liberal”), B) utterances about one’s own identity (e.g., “I’m a Liberal”), C) utterances about others’ identities (e.g., “I know she’s Fascist”), and D) utterances about the current situation (e.g., “we’re in a good condition now”).
