# Supplementary material for: Speech timing cues reveal deceptive speech in social deduction board games
Source: PLoS One. 2022 Feb 11;17(2):e0263852. doi: 10.1371/journal.pone.0263852 (PMC8836341; doi:10.1371/journal.pone.0263852)
Supplement: S4 File — (PDF) [file pone.0263852.s004.pdf]

CAN YOU FIND AND STOP THE...

SECRET HITLER

The year is 1932. The place is pre-WWII Germany. In Secret Hitler, players are German politicians attempting to hold a fragile Liberal government together and stem the rising tide of Fascism. Watch out though—there are secret Fascists among you, and one player is Secret Hitler.

OVERVIEW

At the beginning of the game, each player is secretly assigned to one of three roles: **Liberal**, **Fascist**, or **Hitler**. The Liberals have a majority, but they don't know for sure who anyone is; Fascists must resort to secrecy and sabotage to accomplish their goals. Hitler plays for the Fascist team, and the Fascists know Hitler's identity from the outset, but Hitler doesn't know the Fascists and must work to figure them out.

The Liberals win by enacting five Liberal Policies or killing Hitler. The Fascists win by enacting six Fascist Policies, or if Hitler is elected Chancellor after three Fascist Policies have been enacted.

Whenever a Fascist Policy is enacted, the government becomes more powerful, and the President is granted a single-use power which must be used before the next round can begin. It doesn't matter what team the President is on; in fact, even Liberal players might be tempted to enact a Fascist Policy to gain new powers.

OBJECT

Every player has a secret identity as a member of either the Liberal team or the Fascist team.

Players on the Liberal team win if either:

- Five Liberal Policies are enacted.

OR

- Hitler is assassinated.

Players on the Fascist team win if either:

- Six Fascist Policies are enacted.

OR

- Hitler is elected Chancellor any time after the third Fascist Policy has been enacted.

GAME CONTENTS

17 .... Policy tiles (**6** Liberal, **11** Fascist)
10 .... Secret Role cards
10 .... Party Membership cards
10 .... card envelopes
10 .... Ja! Ballot cards
10 .... Nein Ballot cards
1 .... Election Tracker marker
1 .... Draw pile card
1 .... Discard pile card
3 .... Liberal/Fascist boards
1 .... President placard
1 .... Chancellor placard

SET UP

Select the Fascist track that corresponds to the number of players and place it next to any Liberal track. Shuffle the 11 Fascist Policy tiles and the 6 Liberal Policy tiles into a single Policy deck and place that deck face down on the Draw pile card.

You'll need an envelope for each player, and each envelope should contain a Secret Role card, a corresponding Party Membership card, one Ja! Ballot card, and one Nein Ballot card.
Use the table below to determine the correct distribution of roles.

Liberal Secret Role cards must always be packed together with a Liberal Party Membership card, and Fascist and Hitler Secret Role cards must always be packed together with a Fascist Party Membership card.

# PLAYERS	5	6	7	8	9	10
Liberals	3	4	4	5	5	6
Fascists	1+H	1+H	2+H	2+H	3+H	3+H

Make sure you have the correct number of ordinary Fascists in addition to Hitler!

Once the envelopes have been filled, be sure to shuffle them so each player's role is a secret! Each player should get one envelope selected at random.

WHY ARE THERE SECRET ROLE AND PARTY MEMBERSHIP CARDS?

Secret Hitler features an investigation mechanic that allows some players to find out what team other players are on, and this mechanic only works if Hitler's special role is not revealed. To prevent that from happening, every player has both a Secret Role card and a Party Membership card. Hitler's Party Membership card shows a Fascist party loyalty, but gives no hint about a special role. Liberals who uncover Fascists must work out for themselves whether they've found an ordinary Fascist or their leader.

Once each player has been dealt an envelope, all players should examine their Secret Role cards in secret. Randomly select the first Presidential Candidate and pass that player both the President and Chancellor placards.

GET THE APP: *Go to secrethitler.com/app to get a companion app that can narrate these directions for you.*

For games of 5-6 players, give the following directions to all players:

- *Everybody close your eyes.*
- *Fascist and Hitler, open your eyes and acknowledge each other.*

[Take a long pause]

- *Everyone close your eyes.*
- *Everyone can open your eyes. If anyone is confused or something went wrong, please tell the group now.*

For games of 7-10 players, give the following directions to all players:

- *Everybody close your eyes and extend your hand into a fist in front of you.*
- *All Fascists who are NOT Hitler should open their eyes and acknowledge each other.*
- *Hitler - keep your eyes closed but put your thumb out into a thumbs-up gesture.*
- *Fascists, take note of who has an extended thumb - that player is Hitler.*

[Take a long pause]

- *Everyone close your eyes and put your hands down.*
- *Everyone can open your eyes. If anyone is confused or something went wrong, please tell the group now.*

GAMEPLAY

Secret Hitler is played in rounds. Each round has an **Election** to form a government, a **Legislative Session** to enact a new Policy, and an **Executive Action** to exercise governmental power.

ELECTION

1. **Pass the Presidential Candidacy**
At the beginning of a new round, the President placard moves clockwise to the next player, who is the new Presidential Candidate.

2. **Nominate a Chancellor**
The Presidential Candidate chooses a Chancellor Candidate by passing the Chancellor placard to

any other eligible player. The Presidential Candidate is free to discuss Chancellor options with the table to build consensus and make it more likely the Government gets elected.

Eligibility:
The last *elected* President and Chancellor are "term-limited," and ineligible to be nominated as Chancellor Candidate.

ON ELIGIBILITY:

- *Term limits apply to the President and Chancellor who were last elected, not to the last pair nominated.*

- *Term limits only affect nominations to the Chancellorship; anyone can be President, even someone who was just Chancellor.*

- *If there are only five players left in the game, only the last elected Chancellor is ineligible to be Chancellor Candidate; the last President may be nominated.*

- *There are some other rules that affect eligibility in specific ways: the Veto Power and the Election Tracker. You don't need to worry about those yet, and we'll talk about each one in its relevant section.*

3. **Vote on the government**
Once the Presidential Candidate has chosen an eligible Chancellor Candidate, players may discuss the proposed government until everyone is ready to vote. Every player, *including the Candidates*, votes on the proposed government. Once everyone is ready to vote, reveal your Ballot cards simultaneously so that everyone's vote is public.

If the vote is a tie, or if a majority of players votes no:

The vote fails. The Presidential Candidate misses this chance to be elected, and the President placard moves clockwise to the next player. The Election Tracker is advanced by one Election.

Election Tracker: If the group rejects three governments in a row, the country is thrown into chaos. Immediately reveal the Policy on top of the Policy deck and enact it. Any power granted by this Policy is ignored, but the Election Tracker resets, and existing term-limits are forgotten. All players become eligible to hold the office of Chancellor for the next Election. If there are fewer than three tiles remaining in the Policy deck at this point, shuffle them with the Discard pile to create a new Policy deck.

Any time a new Policy tile is played face-up, the Election Tracker is reset, whether it was enacted by an elected government or enacted by the frustrated populace.

If a majority of players votes yes:

The Presidential Candidate and Chancellor Candidate become the new President and Chancellor, respectively.

▶ **If three or more Fascist Policies have been enacted already:**

Ask if the new Chancellor is Hitler. If so, the game is over and the Fascists win. Otherwise, other players know for sure the Chancellor is not Hitler.

Proceed as usual to the Legislative Session.

LEGISLATIVE SESSION

During the Legislative Session, the President and Chancellor work together to enact a new Policy in secret. The President draws the top three tiles from the Policy deck, looks at them in secret, and discards one tile face down into the Discard pile. The remaining two tiles go to the Chancellor, who looks in secret, discards one Policy tile face down, and enacts the remaining Policy by placing the tile face up on the corresponding track.

Verbal and nonverbal communication between the President and Chancellor is forbidden. The President and Chancellor MAY NOT pick Policies to play at random, shuffle the tiles before discarding one, or do anything else clever to avoid secretly and intentionally selecting a Policy. Additionally, the President should hand both Policies over at the same time, rather than one at a time to gauge the Chancellor's reaction. Attempting to telegraph the contents of your hand using randomness or any other unusual selection procedure violates the spirit of the game. Don't do it.

Discarded Policy tiles should never be revealed to the group. Players must rely on the word of the President and Chancellor, who are free to lie.

ABOUT LYING: Often, some players learn things that the rest of the players don't know, like when the President and Chancellor get to see Policy tiles, or when a President uses the Investigate power to see someone's Party Membership card. You can always lie about hidden knowledge in Secret Hitler. The only time players MUST tell the truth is in game-ending, Hitler-related scenarios: a player who is Hitler must say so if assassinated or if elected Chancellor after three Fascist Policies have been enacted.

If there are fewer than three tiles remaining in the Policy deck at the end of a Legislative Session, shuffle them with the Discard pile to create a new Policy deck. Unused Policy tiles should never be revealed, and they should not be simply placed on top of the new Policy deck.

If the government enacted a Fascist Policy that covered up a Presidential Power, the sitting President gets to use that power. Proceed to the Executive Action.

If the government enacted a Liberal Policy or a Fascist Policy that grants no Presidential Power, begin a new round with a new Election.

EXECUTIVE ACTION

If the newly-enacted Fascist Policy grants a Presidential Power, the President must use it before the next round can begin. Before using a power, the President is free to discuss the issue with other players, but ultimately the President gets to decide how and when the power is used. Gameplay cannot continue until the President uses the power. Presidential Powers are used only once; they don't stack or roll over to future turns.

PRESIDENTIAL POWERS

Investigate Loyalty

The President chooses a player to investigate. Investigated players should hand their Party Membership card (not Secret Role card!) to the President, who checks the player's loyalty in secret and then returns the card to the player. The President may share (or lie about!) the results of their investigation at their discretion. No player may be investigated twice in the same game.

Call Special Election

The President chooses any other player at the table to be the next Presidential Candidate by passing that player the President placard. Any player can become President—even players that are term-limited. The new President nominates an eligible player as Chancellor Candidate and the Election proceeds as usual.

A Special Election does not skip any players. After a Special Election, the President placard returns to the left of the President who enacted the Special Election.

If the President passes the presidency to the next player in the rotation, that player would get to run for President twice in a row: once for the Special Election and once for their normal shift in the Presidential rotation.

Policy Peek

The President secretly looks at the top three tiles in the Policy deck and then returns them to the top of the deck without changing the order.

Execution

The President executes one player at the table by saying "I formally execute [player name]." If that player is Hitler, the game ends in a Liberal victory. If the executed player is not Hitler, the table should **not** learn whether a Fascist or a Liberal has been killed; players must try to work out for themselves the new table balance. Executed players are removed from the game and may not speak, vote, or run for office.

VETO POWER

The Veto Power is a special rule that comes into effect after five Fascist Policies have been enacted. For all Legislative Sessions after the fifth Fascist Policy is enacted, the Executive branch gains a *permanent new ability* to discard all three Policy tiles if **both** the Chancellor and President agree.

The President draws three Policy tiles, discards one, and passes the remaining two to the Chancellor as usual. Then Chancellor may, instead of enacting either Policy, say "I wish to veto this agenda." If the President consents by saying, "I agree to the veto," both Policies are discarded and the President placard passes to the left as usual. If the President does not consent, the Chancellor must enact a Policy as normal.

Each use of the Veto Power represents an inactive government and advances the Election Tracker by one.

STRATEGY NOTES

- **Everyone should claim to be a Liberal.** Since the Liberal team has a voting majority, it can easily shut out any player claiming to be a Fascist. As a Fascist, there is no advantage to outing yourself to the majority. Additionally, Liberals should usually tell the truth. Liberals are trying to figure out the game like a puzzle, so lying can put their team at a significant disadvantage.

- **If this is your first time playing Hitler, just remember: be as Liberal as possible.** Enact Liberal Policies. Vote for Liberal governments. Kiss babies. Trust your fellow Fascists to create opportunities for you to enact Liberal Policies and to advance Fascism on their turns. The Fascists win by subtly manipulating the table and waiting for the right cover to enact Fascist Policies, not by overtly playing as evil.

- **Liberals frequently benefit from slowing play down and discussing the available information.** Fascists frequently benefit from rushing votes and creating confusion.

- **Fascists most often win by electing Hitler, not by enacting six Policies!** Electing Hitler isn't an optional or secondary win condition, it's the core of a successful Fascist strategy. Hitler should always play as a Liberal, and should generally avoid lying or getting into fights and disagreements with other players. When the time comes, Hitler needs the Liberals' trust to get elected. Even if Hitler isn't ultimately elected, the distrust sown among Liberals is key to getting Fascists elected late in the game.

- **Ask other players to explain why they took an action.** This is especially important with Presidential Powers—in fact, ask ahead of time whom a candidate is thinking of investigating/appointing/assassinating.

- **If a Fascist Policy comes up, there are only three possible culprits: The President, the Chancellor, or the Policy Deck.** Try to figure out who (or what!) put you in this position.

THANK YOU

Mike, Tommy, and Mac would like to thank:

Shari Spiro, Dan Shapiro, Elan Lee, Mike Selinker, Luke Crane and everyone else at Kickstarter, everyone who contributed a quote to the Kickstarter, The Cards Against Humanity staff.

Thanks to our early playtesters: Karlee, Tom, Maria, Trin, Andy, Cory, Katie, Veronica, Sandy, Greg, Andrew, and everyone else who helped us refine the earliest versions of the game.

Most especially, thank you to our 34,565 backers who helped us make this game a reality.

Secret Hitler was funded with **Kickstarter**.

CREDITS & LICENSE

Secret Hitler was created by Mike Boxleiter, Tommy Maranges, and Mac Schubert.

Secret Hitler is licensed under a Creative Commons Attribution-NonCommercial-ShareAlike 4.0 International License.

YOU ARE FREE TO:

- **Share** – copy and redistribute the game in any medium or format
- **Adapt** – remix, transform, and build upon the game

UNDER THE FOLLOWING TERMS:

- **Attribution** – If you make something using our game, you need to give us credit and link back to us, and you need to explain what you changed.
- **Non-Commercial** – You can't use our game to make money.
- **Share Alike** – If you remix, transform, or build upon our game, you have to release your work under the same Creative Commons license that we use (BY-NC-SA 4.0).
- **No additional restrictions** – You can't apply legal terms or technological measures to your work that legally restrict others from doing anything our license allows. That means you can't submit anything using our game to any app store without our approval.

You can learn more about Creative Commons at CreativeCommons.org. (Our license is available at CreativeCommons.org/licenses/by-nc-sa/4.0/legalcode).
